# Magnitude and associated factors of unplanned extubation in intensive care unit: A multi-center prospective observational study

**DOI:** 10.1016/j.amsu.2022.103936

**Published:** 2022-06-08

**Authors:** Zewdu Minda, Hirbo Samuel, Senayit Aweke, Getachew Mekete, Abdurehman Seid, Denberu Eshetie

**Affiliations:** a Department of Anesthesia, Saint Pitter Specialized Hospital, Addis Ababa, Ethiopia; b Department of Anesthesia, Addis Ababa University, School of Medicine, Addis Ababa, Ethiopia; c Department of Anesthesia, College of Health Sciences, School of Medicine, Debre Tabor University, Debre Tabor, Ethiopia; d Department of Public Health, Saint Pitter Specialized Hospital, Addis Ababa, Ethiopia

**Keywords:** Unplanned extubation, Magnitude, Risk factor, Ethiopia, AOR, Adjusted Odd Ratio, ASA, American Society of Anesthesiologists, C.I, Confidence interval, COR, Crude odd ratio, BSA, Body Surface Area, ENT, Ear Nose Throat, GA, General Anesthesia, GIT, Gastrointestinal Tracts, PACU, Post Anesthesia Care Unit, SPSS, Statistical Package for Social Sciences

## Abstract

**Background:**

Unplanned extubation is the removal of an endotracheal tube accidently during procedural activities or by the action of the patient. It is one of the commonly reported complications among mechanically ventilated patients in the intensive care unit. This study aimed to assess the magnitude and associated factors of unplanned extubation in intensive care units at referral hospitals in Addis Ababa, Ethiopia, 2021.

**Methods:**

Institutional based prospective observational study was conducted on 317 intubated patients in the intensive care unit at referral hospitals of Addis Ababa, Ethiopia, from January 8, 2021–May 9, 2021. Data were collected using a structured questionnaire. Descriptive statics were expressed in percentages and presented with tables and figures. Both Bivariable and multivariable logistic analysis was done to identify factors associated with unplanned extubation in intensive care unit. P < 0.05 with 95% CI was set as Statistical significance.

**Result:**

The prevalence of unplanned extubation in this study was 19.74%. Being male (AOR = 3.132, 95%CI: 1.276–7.69), duration of intubation <5days (AOR = 2.475, 95% CI: 1.039–5.894), managed by junior resident (AOR = 5.25, 95% CI: 2.125–12.969), being physically restrained (AOR = 4.356, 95%CI: 1.786–10.624), night shift (AOR = 3.282, 95%CI:1.451–7.424)and agitation (AOR = 4.934,95%CI:1.934–12.586) were significantly contribute to the occurrence of unplanned extubation.

**Conclusion:**

and recommendation: This study showed that the prevalence of unplanned extubation was high in the intensive care unit. We suggest to intensive care unit staff to give special attention to early intubated patients, especially male individuals and the stakeholders of hospitals should rearrange the time of shift and physician schedules in the intensive care unit.

## Highlights

• Magnitude of unplanned extubation was high in the intensive care unit.
• Duration of intubation less than 5 days was significantly associated.
• Give special attention to early intubated patients and rearrange the time of shift.

## 1 Introduction

Endotracheal tube (ETT) intubation is one of the lifesaving procedures performed in intensive care units (ICU)when the patient required respiratory support [1]. Maintaining endotracheal intubation is the first and most priority important factor in mechanically ventilated patients to save their lives and maintaining ventilation through the artificial airway in intensive care [2]. These airway management-related complications end up with life-threatening conditions like hypoventilation and hypoxia [3]. Unplanned extubation is the premature removal of ETT accidently during medical care or by the action of the patient [4].

Prevention of unplanned extubation is important since it may lead to serious complications, like airway trauma, compromised hemodynamic and respiration [5], prolonged duration of mechanical ventilation, and prolonged hospital stay [6,7], which leads to high mortality, morbidity and inefficient use of resource [8]. The incidence of reintubation and cardiovascular accidents after unplanned extubation were 61.1% and 8.33% respectively in Philippines general hospitals [9].

The incidences of unplanned extubation in developed countries vary with different ICU settings and have remained unchanged with rates ranging from 0.5% to 38.5% [10]. But there are limited data sources in developing countries about the incidence of unplanned extubation. The incidence of UE in South Africa in 2004 in the academic intensive care unit and Egypt Zigzag University respiratory ICU in 2019 was 10.3% [11] and 11.02% [12] respectively.

Factors predisposing to the occurrence of unplanned extubation can be patient-related, ICU staff-related, activity-related, and time of shift-related factors. The incidence of UE is highest in male individuals [13]. Intubated patients who are physically restrained and less sedated are at high risk for the occurrence of unplanned extubation [14].

The study was aimed to assess the magnitude and associated factors of unplanned extubation in intensive care units at referral hospitals in the capital city of Ethiopia.

## 2 Methods and materials

### 2.1 Study area

This prospective observational study was conducted in the capital city of Ethiopia, Addis Ababa in public referral hospitals. The altitude of the city ranges from 2200 to 3000 m above sea level with an average temperature of 22.8 °C. According to the 2021 census, about 5005524 people live in the city. There are 40 hospitals, 29 health Centers, 122 health stations, 37 health posts, and 382 modern private clinics in Addis Ababa. The study was done in selected governmental hospitals in the city which give intensive care unit services. There are 13 public hospitals and around 98 health centers in this city. Among 13 hospitals 9of those give normal ICU service, and one hospital serves as a COVID-19 center since March 2020. This study is registered at researchregistry7855. Also, it is reported according to STROCSS criteria [15].

### 2.2 Study design

An institutional-based multicenter prospective observational study was conducted on intubated patients at the intensive care unit in referral hospitals of Addis Ababa, Ethiopia from January 8/2021 up to May 9/2021.

### 2.3 Source population

All intubated patients in ICU in referral hospitals of Addis Ababa, Ethiopia were the source population.

### 2.4 Study population

All intubated ICU patients during the study period at selected Addis Ababa government hospitals.

### 2.5 Inclusion and exclusion criteria

All intubated patients in ICU at the time of the study period were included in the study and all intubated patients in I CU with COVID- 19 were excluded from the study.

### 2.6 Dependent Variable

Unplanned extubation.

### 2.7 Independent Variables

**Socio-demographic characteristics**: Age, sex.

**Patient factors**: GCS, RASS, physical restraint, previous history of unplanned extubation.

**Staff related factors**: nurse: patient ratio, experience, presence or absence of staff during extubation.

**Activities related factors**: Type of airway securing.

**Shifting time-related factors**: morning, afternoon, night.

**Sedative related factors**: benzodiazepine, ketamine, propofol.

### 2.8 Sample size and sampling technique

A simple random sampling technique was used to select the hospitals by using the lottery method. Five hospitals were selected among 9 hospitals which were Tikur Anbesa specialized hospital (TASH), Saint Paul millennium hospital (SPMH), Zewditu memorial hospital(ZMH), Yekatit 12 hospital, and Minillik second hospital. All 317 patients in these selected governmental hospitals intubated in ICU from January 8, 2021–May 9, 2021 were included in the study.

### 2.9 Data collection techniques

Data was collected by using an English version structured questionnaire taken from studies and data collection was done by trained data collectors. The process of data collection was monitored by the principal investigator. During the process, the principal investigator also stayed close with the data collectors to help when they face getting any problems or faced difficulties.

Demographic data collected was age and sex during preparation of intubation. Under intubation state of the patient route of admission to ICU, reason of intubation, route of intubation, and the type of securing material to fix the ETT were collected. When the patients were extubated, RASS before extubation, GCS before extubation, the drugs used within 24hrs before extubation occurred, the experience of the caregivers' physician, whether the patient was physically restrained or not before extubation, nurse to patient ratio and time of extubation were collected, additionally if the extubation was unplanned; location of the patient during extubation, the situation when extubation occurred, staff at bedside or not during extubation, were immediately collected by the responsible data collector.

### 2.10 Data quality assurance

After training was given to data collectors, data were collected and properly filled in the prepared format. The supervision was made throughout the data collection period to make sure the accuracy, clarity, and consistency of the collected data.

### 2.11 Data analysis and interpretation

The collected data were coded and entered into Epi data 4.6.0.2 and exported to SPSS version 26 statistical software for further analysis. Before starting the analysis recording was done on some of the variables. All independent variables with the dependent variable were analyzed by using binary logistic regression to identify variables that were predictive of the dependent variable. Odd ratio, 95% confidence interval, and P-value were computed to differentiate the risk factors and to assess the strength of association. Variables with a P-value less than 0.25 on binary logistic regression analysis were going to multivariable logistic regression analysis and the cutting point to test the statistical significance was P-value less than 0.05.

### 2.12 Ethical considerations

The study was done after obtaining ethical clearance from the Addis Ababa University College of health science department of anesthesia ethical committee. A formal letter was submitted to each selected hospital from the department of anesthesia and permission was assured to keep the confidentiality. Written consent was obtained from a family member or attendant of intubated patients. The data collection process was held within the selected hospital and the study participant's family or attendant was informed before starting the data collection about the whole thing and the benefit and risks of participating in the study.

## 3 Result

### 3.1 Socio-demographic characteristics of the study participants

A total of 317patients were intubated in ICU during the study period. Three patients were excluded from the study due to incomplete data. Out of 317 intubated patients in ICU 314 were included in the study with a response rate of 99.05% and the median age of the patients was 40. The majority of the patients intubated in the ICU included in the study were male (55.7%) Table 1.

**Table 1:** Socio-demographic characteristics of study participant patients intubated in ICU (n = 314).

variables	Variable category	frequency	percent
Sex	Female	139	44.3
	Male	175	55.7
Age	0–14	37	11.8
	15–47	145	46.2
	48–63	66	21.0
	>64	66	21.0

Intubation states of the study participants.

The majority of patients intubated in ICU were admitted from emergency 102 (32.5%), 127(40.4%) intubated for the indication of respiratory problems, 312(99.4%) patients were intubated orally and 306(97.5%) intubated with cuffed ETT Table 2.

**Table 2:** Intubation states of study participant patients intubated in ICU (n = 314).

Variable	Category variable	frequency	percent
**Route of admission**	from ED	102	32.5
	from ward	97	30.9
	from OT	67	21.3
	from other hospital	48	15.3
**Cause of intubation**	respiratory distress(air way protection)	127	40.4
	septic shock	59	18.8
	neurovascular	16	5.1
	trauma	76	24.2
	cardio vascular	20	6.4
	endocrine	7	2.2
	kidney	9	2.9
**Route of intubation**	oral	312	99.4
	nasal	2	.6
**Type of ETT**	cuffed	306	97.5
	Un cuffed	8	2.5
**Plaster ETT Fixation**	No	247	78.7
	Yes	67	21.3
**Roll bandage ETT fixation**	No	191	60.8
	Yes	123	39.2
**Roll bandage + plaster ETT fixation**	No	177	56.4
	Yes	137	43.6

### 3.2 Risk factors for unplanned extubation for study participant patients intubated in ICU

Majority of patients 96.5% had no previous history of intubation, 127(40.4%) were with GCS 9T, 108(34.4%) patients were sedated with ketamine, 127(40.4%) had 5–10 days duration of intubation, 138(43.9%) patients were physically restrained and 138(38.5%) patients were extubated at night shift Table 3.

**Table 3:** Risk factors for unplanned extubation for study participant patients intubated in ICU (n = 314).

Variables	Category	Frequency	Percent
**History of intubation**	No	303	96.5
	Yes	11	3.5
		
**GCS**	10T	54	17.2
	9T	127	40.4
	8T	60	19.1
	<8T	73	23.2
**Nurse:patient ratio**	1:1	155	49.4
	1:2	159	50.6
**Drug**	benzodiazepine	104	33.1
	ketamine	108	34.4
	propofol	51	16.2
	ketamine + benzodiazepine	51	16.2
**Physician**	junior resident	152	48.4
	senior resident	162	51.6
**Agitation**	No	236	75.2
	Yes	78	24.8
**Very agitated**	No	304	96.8
	Yes	10	3.2
**Drowsy agitated**	No	291	92.7
	Yes	23	7.3
**Restless**	No	266	84.7
	Yes	48	15.3
**Lightly sedated**	No	274	87.3
	Yes	40	12.7
**Alert& calm**	No	185	58.9
	Yes	129	41.1
**<5 days of intubation**	No	209	66.6
	Yes	105	33.4
**5**–**10 days of intubation**	No	187	59.6
	Yes	127	40.4
**>10days of intubation**	No	222	70.7
	Yes	92	29.3
**Physically restrained**	No	176	56.1
	Yes	138	43.9
**Anesthetist (anesthesiologist)**	No	156	49.7
	Yes	158	50.3
**Extubation at morning**	No	210	66.9
	Yes	104	33.1
**Extubation at afternoon**	No	224	71.3
	Yes	90	28.7
**Extubation at night**	No	193	61.5
	Yes	121	38.5

### 3.3 Magnitude of unplanned extubation in ICU

**Fig. 1:**
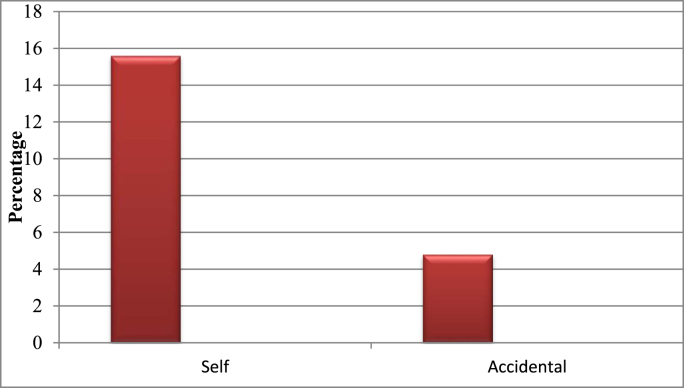
Types of unplanned extubations occurred in ICU at selected Addis Ababa governmental hospitals, Addis Ababa, Ethiopia from January 8, 2021–May 9, 2021.

**Fig. 2:**
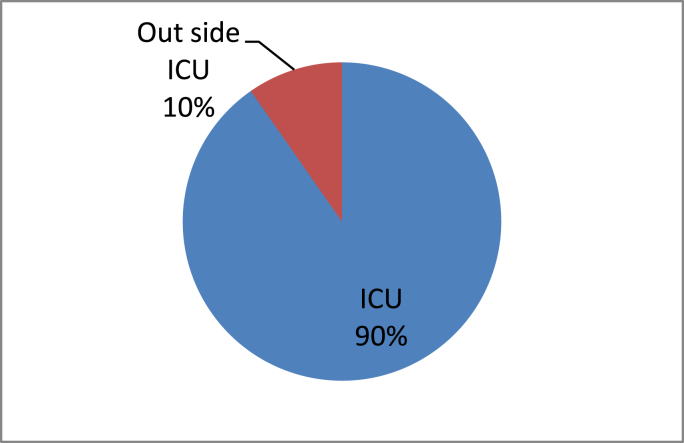
Place of unplanned extubation occurrence among patients intubated in ICU at selected Addis Ababa governmental hospitals, Addis Ababa, Ethiopia from January 8, 2021–May 9, 2021.

In this study 62(19.74%) patients experienced unplanned extubation. Five (1.6%) patients experienced unplanned extubation twice. There was 67 episode of unplanned extubation. Of these 49(15.6%) were self -extubation and (15) 4.8% were accidental extubation. Fifty-six patients experienced unplanned extubation in ICU which was 90.3% of unplanned extubation occurred in ICU and the remaining 6(9.7%) patients’ experienced unplanned extubation outside ICU Figs. 1 and 2.

**Fig. 3:**
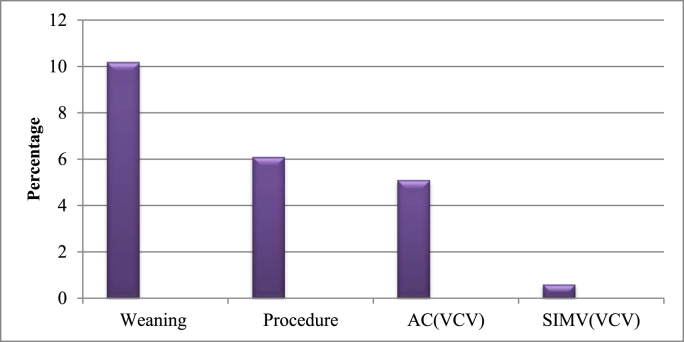
Distribution of unplanned extubation occurred at selected Addis Ababa governmental hospitals depending on situation and mode of ventilation, Addis Ababa, Ethiopia from January 8, 2021–May 9, 2021.

On the other hand, 47(15%) unplanned extubations occurred when clinical staff at the bed and 15(4.7%) occurred in the absence of staff. Thirty-two (10.2%) unplanned extubations occurred during weaning, nineteen (6.1%) during procedural activities, sixteen (5.1%) during mechanical ventilation with assisted control volume control ventilation (ACVCV) mode, and two (0.6%) were during mechanical with synchronized intermittent mechanical ventilation volume control ventilation (SIMV VCV) mode Fig. 3.

### 3.4 Factors associated with unplanned extubation

With adjustment to other variables, being male, intubation duration <5days, patients managed by a junior resident, agitation, being physically restrained and night shift were significantly associated with unplanned extubation (Table 4).

**Table 4:** Factors associated with unplanned extubation in ICU intubated patients (n = 314).

Variables	Category	Unplanned extubation		COR(95%CI)	AOR (95%)	P-Value
		Yes n (%)	No n (%)			
Sex	Female	17(5.41)	122(38.85)	1	1	0.013
	Male	45(14.33)	130(41.40)	2.484(1.349–4.573)	3.132(1.276–7.691)*	
Age	0–14	3(1)	34(10.82)	0.360(0.095–1.356)	0.605(0.095–3.844)	0.594
	15–47	37(11.78)	108(34.39)	1.397(0 .685–2.847)	1.84(0.67–5.091)	0.239
	48–63	9(2.9)	57(18.15)	0.644(0.254–1.629)	0.765(0.216–2.706)	0.677
	>64	13(4.14)	53(16.9)	1	1	
Route admission	From ED	28(8.92)	74(23.6)	1.829(0.767–4.36)	0.1.215(0.378–3.904)	0.743
	From ward	14(4.46)	84(26.4)	0.512(0.86–1.408)	0.552(0.142–2.152)	0.392
	From OT	11(3.5)	56(17.83)	0.759(0.273–2.110)	0.81(0.208–3.145)	0.76
	From other hospital	9(2.9)	39(12.42)	1	1	
GCS	10T	12(3.8)	42(13.4)	1.800(0.713–4.542)	1.269(0.34–4.73)	0.722
	9T	38(12.1)	89(28.3)	2.69(1.248–5.796)	1.86(0.62–5.585)	0.27
	8T	2(0.6)	58(18.5)	0.217(0.046–1.033)	0.41(0.059–2.83)	0.366
	>8T	10(3.2)	63(20)	1		
Drug	Benzodiazepine	34(10.83)	70(22.3)	1.991(0.892–4.448)	1.447(0.451–4.641)	0.534
	Ketamine	9(2.9)	98(31.2)	0.373(0.141–0.985)	0.338(0.090–1.276)	0.109
	Propofol	7(2.2)	43(13.7)	0.879(0.324–2.384)	0.667(0.153–2.921)	0.591
	Ketamine benzodiazepine	12(3.8)	41(13.05)	1	1	
Physician	Junior resident	49(15.6)	95(30.25)	5.544(1.638–5.38)	5.890(2.344–14.800)*	0.000
	Senior resident	13(4.1)	157(50)	1	1	
Previous intubation	No	56(17.83)	247(78.7)	1	1	0.148
	Yes	6(1.9)	5(1.6)	1.926(0.796–4.66)	2.551(0.717–9.085)	
<5days intubation	No	24(7.6)	185(58.9)	1	1	0.028
	Yes	38(12.1)	67(21.3)	4.372(2.44–7.82)	2.659(1.110–6.370)*	
>10days intubation	No	53(16.9)	169(53.82)	1	1	0.938
	Yes	9(2.8)	83(26.43)	0.346(0.163–0.735)	0.957(0.322–2.843)	
Agitation	No	28(8.9)	208(66.24)	1	1	0.001
	Yes	34(10.82)	44(14)	5.740(3.161–10.42)	5.014(1.992–12.621)*	
Restless	No	48(15.3)	218(69.42)	1	1	0.159
	Yes	14(4.41)	34(10.82)	1.870(0.932–3.753)	2.130(0.744–6.094)	
Anesthetist (anesthesiologist)	No	40(12.74)	102(32.48)	1	1	0.414
	Yes	22(7)	150(47.8)	1.654(0.941–2.909)	0.715(0.320–1.598)	
Physically restrained	No	15(4.7)	161(51.27)	1	1	0.001
	Yes	47(15)	91(29)	5.544(2.936–10.47)	4.352(1.788–10.596)*	
Night shift	No	23(7.32)	170(54.14)	1	1	0.004
	Yes	39(12.42)	82(26.1)	3.515(1.97–6.27)	3.307(1.461–7.487)*	
Roll bandage EET fixation	No	46(14.6)	145(46.17)	1	1	0.218
	Yes	16(5.1)	107(34.07)	0.471(0.253–0.88)	0.580(0.244–1.380)	

Note: 1 = Reference group COR=Crude odd ratio.

n = number of patient AOR = Adjusted odd ratio.

% = percent CI=Confident interval.

In the study patients who were male were three times more likely to face unplanned extubation than females. (Table 4).

Patients managed by junior residents were more than five times more likely to experience unplanned extubation than senior residents (Table 4) and patients intubated for less than five days were more than two times more likely to experience unplanned extubation(Table 4).

On other occasions patients who were physically restrained more than four times likely increase the occurrence of unplanned extubation (Table 4).

The result of this study suggested that patients who were agitated more than four times increase the occurrence of unplanned extubation(Table 4).

This study showed that the odd of the night shift was 3.28 times more likely associated with the occurrence of unplanned extubation (Table 4).

## 4 Discussion

Unplanned extubation was the removal of an endotracheal tube accidental or by the action of the patient [16]. In this study, the magnitude of unplanned extubation was 19.74% which was in the range of 0.5%–35.8% reported in the systematic literature review [10]. The result we found on the magnitude of unplanned extubation was in line with the study which was done in the Philippines, unplanned extubation rate of 19% [9], and the research done in Taiwan with an incidence rate of 22.5% [17]. On the other hand, the result was inconsistent with research done in British (4.47%), Belgium (4.2%), and Spain (10%) [18]. Also in this study, the magnitude of unplanned extubation was higher than a study reported by most studies [19]. The possible reason for this Addis Ababa governmental hospitals are the area of trainee and was loaded with junior resident which has a significant contribution to the incidence of unplanned extubation evidenced by our data. This was supported by the research done in Egypt in 2019 patients managed by junior residents were more likely to increase the probability of unplanned extubation [9]. The other reason most of them used a case-control study design,cross-sectional study design, they were get secondary data from previous records which has the chance of losing the case. This high value in this study may be due to the study area, most of them were done in single study area.

In our study, the majority of unplanned extubation were self-extubation (88.05%) which was in line with a study done in Malaysia(1) and South Africa(4). Also, most, of the unplanned extubation occurred when clinical staff at the bedside in ICU which was the same as the research done in Malaysia(1). Of a total of unplanned extubation (19.74%) in this study, the result showed that 10.2% occurred during the weaning process. The possible justification for this during the weaning process was an inappropriate reduction of sedative drugs and a decreased concentration of caregivers due to the patient becomes more stable than at the beginning of the patient's condition.

The result of this study showed being male was a risk factor for the occurrence of unplanned extubation. Male patients are three times more likely to experience unplanned extubation than females (Table 4). The reason for this could be male patients are stronger than females due to the overall increase of muscle mass in the male by the effect of testosterone [19]. This could result in removing the ETT by themselves. This result was similar to different studies [11]. But another study reported in 2016 by Aydog.S et al. being male was not significantly associated with the incidence of unplanned extubation [16].

The patient managed by junior residents were more than five times more likely associated with the occurrence of unplanned extubation than senior residents (Table 4). This result was supported by Abbas et al., in 2019 [18]. The reason for this could be junior residents are still learning and demanded high workload and stress than senior residents. The other could be they have less skill in the management of patients in the intensive care unit.

Another significant association with the occurrence of unplanned extubation was the intubation duration of fewer than five days. We found patient intubated for less than five days was two times more likely to experience unplanned extubation than others (Table 4). This was supported by authors Chin Dc et al.(1) and Gueret.RM et al. [20]. The possible justification would-be patients within these days wouldn't adapt to the ETT, feeling discomfort, and remove the ETT by themselves as most of the unplanned extubation was self-extubation (15.6%) evidenced by our data.

We found in this study higher level of consciousness or GCS greater than 9T was not associated with the occurrence of unplanned extubation. In contrast to this different studies showed a higher level of consciousness GCS greater than 9T was associated with the occurrence of unplanned extubation [21]. The possible reason for this discrepancy could be patients whose levels of consciousness higher or GCS greater than 9T were had a high level of sedation.

In this study, agitation was associated with the incidence of unplanned extubation (Table 4). This finding was supported by different studies done in different countries [22]. This could be because when patients are agitated they became unstable and experience unnecessary movement. This movement leads to the removal of ETT during medical care accidently or by the action of the patient.

Physically restrained was significantly associated with the occurrence of unplanned extubation. Patients who were physically restrained were more than four times more likely to experience unplanned extubation than those not physically restrained (Table 4). This finding was in agreement with different studies [23]. The possible justification could be physically restrained may increase anxiety due to the inability to express themselves by moving their hand freely and gestures. The other reason could be restraining the patients may be considered as the preventive strategy of unplanned extubation in agitated patients without appropriate sedation protocol.

We found in this study night shift had a significant association with the incidence of unplanned extubation (Table 4). This finding was supported by authors Kwon E et al., in 2017 and Abbas A et al., in 2019 [16]. The reason for this could be staff assigned for the night shift are working for a long time than staff working the morning shift and afternoon shift. Staff working during the night shift may spend day time by other work and become exhausted during the night time may be the other reason. In addition to the above, the biological sleep –awake may play an important role, during night time the patient may be disturbed by the alarming of the ventilator and monitoring. The staffs may also become sleepy due to this biological sleep-wake cycle.

## 5 Conclusions

The prevalence of unplanned extubation was 19.74% in Addis Ababa governmental hospitals which was high compared to most studies conducted worldwide and also conducted in Africa. Being male, patients managed by a junior resident, intubation duration less than five days, agitation, being physically restrained, and night shift were significantly associated with the occurrence of unplanned extubation.

## Recommendations

The hospital management and ICU staffs in Addis Ababa governmental hospitals should minimize the incidence of unplanned extubation in ICU by preparing appropriate sedation protocol, rearrangement shifting time, and programs for ICU staffs.

## Limitation of the study

The limitation of this study was we did not include COVID-19 ICU due to precaution issues during data collection time, small sample size, and the duration of follow-up was short so that we did not study complications of reintubation.

## Authors' contributions

All authors contributed to the inception, design, analysis, interpretation, and drafting of the research manuscript. Also, all authors read and approved the revised manuscript for publication.

## Ethics approval and consent

Ethical clearance was obtained from Addis Ababa University institutional review board and an Official support letter was given from referral hospitals of Addis Ababa as well informed written consent was secured from each study participant's family. Confidentiality was assured throughout the research.

## Consent for publication

Not applicable.

## Availability of data and material

The data of this study will be available from the corresponding author on reasonable request.

## Funding

Not funded

## Provenance and peer review

Not commissioned, externally peer-reviewed.

## Registration of research studies

1. Name of the registry: http://www.researchregistry.com.

2. Unique Identifying number or registration ID: researchregistry7644.

3. Hyperlink to your specific registration (must be publicly accessible and will be checked): https://www.researchregistry.com/browse-the-registry#home/registrationdetails/626c4416f9d2c7001e1714ea/

## Guarantor

The Guarantor is the one or more people who accept full responsibility for the work and/or the conduct of the study, had access to the data, and controlled the decision to publish.

## Declaration of competing interest

The authors declare there is no competing interest in this work.
